# miR‐221‐3p targets Ang‐2 to inhibit the transformation of HCMECs to tip cells

**DOI:** 10.1111/jcmm.17892

**Published:** 2023-07-31

**Authors:** Peng Yang, Qing Yang, Yiheng Yang, Qingshan Tian, Zhenzhong Zheng

**Affiliations:** ^1^ Department of Cardiology The First Affiliated Hospital of Nanchang University Nanchang China; ^2^ Department of Cardiology Gaoxin Branch of The First Affiliated Hospital of Nanchang university Nanchang China; ^3^ Jiangxi Hypertension Research Institute Nanchang China

**Keywords:** Ang‐2, angiogenesis, HCMECs, miR‐221‐3p, tip cell

## Abstract

Postembryonic angiogenesis is mainly induced by various proangiogenic factors derived from the original vascular network. Previous studies have shown that the role of Ang‐2 in angiogenesis is controversial. Tip cells play a vanguard role in angiogenesis and exhibit a transdifferentiated phenotype under the action of angiogenic factors. However, whether Ang‐2 promotes the transformation of endothelial cells to tip cells remains unknown. Our study found that miR‐221‐3p was highly expressed in HCMECs cultured for 4 h under hypoxic conditions (1% O_2_). Moreover, miR‐221‐3p overexpression inhibited HCMECs proliferation and tube formation, which may play an important role in hypoxia‐induced angiogenesis. By target gene prediction, we further demonstrated that Ang‐2 was a downstream target of miR‐221‐3p and miR‐221‐3p overexpression inhibited Ang‐2 expression in HCMECs under hypoxic conditions. Subsequently, qRT‐PCR and western blotting methods were performed to analyse the role of miR‐221‐3p and Ang‐2 on the regulation of tip cell marker genes. MiR‐221‐3p overexpression inhibited CD34, IGF1R, IGF‐2 and VEGFR2 proteins expression while Ang‐2 overexpression induced CD34, IGF1R, IGF‐2 and VEGFR2 expression in HCMECs under hypoxic conditions. In addition, we further confirmed that Ang‐2 played a dominant role in miR‐221‐3p inhibitors promoting the transformation of HCMECs to tip cells by using Ang‐2 shRNA to interfere with miR‐221‐3p inhibitor‐treated HCMECs under hypoxic conditions. Finally, we found that miR‐221‐3p expression was significantly elevated in both serum and myocardial tissue of AMI rats. Hence, our data showed that miR‐221‐3p may inhibit angiogenesis after acute myocardial infarction by targeting Ang‐2 to inhibit the transformation of HCMECs to tip cells.

## 1 INTRODUCTION

Cardiomyocytes need an effective blood supply to meet the high metabolic demands and oxygen demands. Angiogenesis can induce improvements in blood flow, revascularization and myocardial function, and may be a new mechanism to promote cardiac repair and myocardial survival.^1^ In the strictest, angiogenesis means the growth of blood vessels from already existing vessels. Attracted by proangiogenic signals, vascular endothelial cells have the ability to be motor, invasive and prominent filopodia.^2^ The tip cells that compose sprouting blood vessels, like growth cones, guide blood vessels by sensing environmental cues, and are a transdifferentiated phenotype of endothelial cells induced by angiogenic factors.^3^, ^4^ Hypoxia is the main physiological signal of angiogenesis. Angiogenesis is primarily an adaptive response to tissue hypoxia, and can occur during embryonic development or tumour growth when the tissue expansion rate exceeds the formation of high hypoxia conditions. Angiogenesis can also occur in the case of trauma or myocardial ischemia.^5^

MicroRNAs (miRNAs) are a class of endogenous single‐stranded RNA small molecules of approximately 22 nucleotides in length. Although they cannot be further translated into proteins, miRNAs can specifically bind to the target mRNA, thereby indirectly inhibiting the expression of post‐transcriptional genes.^6^ Compared to mRNAs, miRNAs are more stable and resistant to ribonuclease effects, allowing miRNAs to act as biomarkers for certain diseases.^7^, ^8^, ^9^ In recent years, more and more studies have shown that many miRNAs are involved in the regulation of angiogenesis, such as miR‐320,^10^ miR‐92a,^11^ miR‐128,^12^ miR‐126,^13^ miR‐210^14^ and miR‐214.^15^ MiR‐221 is also a miRNA and its gene is located on the x chromosome. After multiple editing modifications and cleavage of the dicer enzyme, the mature body forms and its 3p end comprises miR‐221‐3p.^16^ MiR‐221 is essential for angiogenesis, miR‐221 is required for the proliferation and migration of tip cells, and miR‐221 knockout is associated with defects in vascular endothelial growth factor receptor 3 (VEGFR3) expression in tip cells.^17^ MiRNAs can promote or inhibit angiogenesis after hypoxia, depending on their targets.^18^ A study has shown that hypoxia inducible factor‐1α (HIF‐1α) is the main target gene of miR‐221‐3p in human umbilical vein endothelial cells (HUVECs), and miR‐221‐3p suppresses angiogenesis in endothelial cells by targeting HIF‐1α.^19^ However, thrombospondin‐2 (THBS2) is also a downstream targets of miR‐221‐3p, and miR‐221‐3p promoted trophoblast growth, invasion and migration by targeting THBS2.^20^ Whether there are other downstream targets of miR‐221‐3p is uncertain. Our study aims to further explore novel downstream targets of miR‐221‐3p and the mechanisms involved in angiogenesis.

Angiopoietins are an important family of growth factors. Members of this family include angiopoietin 1(Ang‐1), Ang‐2, Ang‐3 and Ang‐4. The activities of these family members are mediated by the endothelial‐specific receptor tyrosine kinase 1(tie1) and tie2.^21^ Tie2 is the major binding receptor for all angiopoietins in the Ang/tie signalling pathway.^22^ Ang‐1 increases the association of endothelial cells with pericytes and vascular smooth muscle cells to stimulate the maturation and stabilization of sprouting blood vessels.^23^ Although Ang‐1 and Ang‐2 have similar binding affinities for tie2 receptors, they exert opposite effects on tie2 activation. Ang‐2 is upregulated under hypoxic condition,^24^ and acts as an antagonist of the Ang‐1/Tie2 axis, resulting in disruption of endothelial junction integrity and vascular instability.^25^ Moreover, it has been shown that Ang‐2 may inhibit angiogenesis by promoting the shedding of pericytes.^26^ In contrast, Ang‐2 has been found to enhance the sensitivity of vascular endothelial growth factor (VEGF) and to initiate angiogenesis in cooperation with VEGF.^27^ Therefore, the effect of Ang‐2 in angiogenesis is complex as well as requiring further exploration. The transformation of vascular endothelial cells into tip cells is the initiating factor of vascular germination, which is a key process of angiogenesis.^28^ In this study, we first investigated the targeting relationship between miR221‐3p and Ang‐2, and then further explored the role of miR‐221‐3p and Ang‐2 in the transformation of endothelial cells to tip cells. Finally, we constructed Ang‐2 knockdown models by Ang‐2 shRNA for clarifying the role of Ang‐2 in the downstream function of miR‐221‐3p.

## 2 MATERIALS AND METHODS

### 2.1 Animals and grouping

Ten male sprague–dawley (SD) rats (age, 6–8 weeks), weighing 220–250 g, were purchased from Institute of Animal Research in Wuhan University. Rats were randomly assigned to the acute myocardial infarction (AMI) group (*n* = 5) and sham‐operation group (*n* = 5). Animals were kept under standard conditions with a mean temperature of 25 ± 2°C, a mean relative humidity of 50% ±20 and a light–dark cycle of 12:12 h.

### 2.2 Induction of AMI model

The AMI model was constructed by ligating the left anterior descending (LAD) coronary arteries of the rats according to Wang's^29^ methods. The induction of AMI model are briefly described below. Rats were induced and maintained under anaesthesia by intraperitoneal injection of 0.6 mL/kg ketamine and 2.5 mg/kg valium, and connected to the DW‐3000S dual channel small animal ventilator (Anhui Zhenghua Biological Instrument and Equipment Co.). Ventilation indexes were set as follows: Tidal volume: 6–8 mL; The ratio of inhalation to respiration 1:2; Respiration rate: 70 times/min. Then, the rat's extremities were connected to the RM6240E/EC multichannel physiological signal acquisition and processing system (Chengdu Instrument Factory) with pinpoint electrodes to monitor and record the changes observed in electrocardiogram. The animal was placed in the right lateral position and the temperature was monitored and maintained at 37°C. After thoracotomy, the pericardium was opened and the LAD coronary artery was ligated with a 5‐0 suture next to the pulmonary conus inferior to the root of the left appendage. The AMI model was regarded to successfully produced when the left ventricular myocardium changed from red to white, accompanied by diminished apical beats and a significant increase in the ST segment of the electrocardiogram. Asham‐operation group was produced using the same method without ligation of the anterior descending coronary artery. After successful modelling, 0.5 mL of blood was taken from each group through the orbital vein before euthanasia of the rats, which was used to detect the serum level of miR‐221‐3p and miR‐222‐3p by quantitative real‐time polymerase chain reaction (qRT‐PCR) method. After the rats were executed, myocardial tissue was taken from the infarcted area in each group for the detection of miR‐221‐3p and miR‐222‐3p levels in myocardial tissue by qRT‐PCR method. The protocol of this study was approved by the Ethics Committee of The First Affiliated Hospital of Nanchang University (IACUC number: 202205QR006).

### 2.3 Biological pathway analysis and target prediction

The target genes of miR‐221‐3p that have been verified in published studies were collected and then enriched using Funrich3.13 software (http://www.funrich.org/). TargetScan7.2 (http://www.targetscan.org/) was used to predict miR‐221‐3p targeting Ang‐2 and the binding regions.

### 2.4 Cell cultures

Human cardiac microvascular endothelial cells (HCMECs, ScienCell, Cat. No. 6000) purchased from ScienCell Research Laboratories. HCMECs were cultured in endothelial cell medium (ECM, ScienCell, Cat. No. 1001) containing 5% fetal bovine serum (FBS, Zhengbo Weiye, Cat. No. YTYS1050), 1% endothelial cell growth supplement (ECGS, ScienCell, Cat. No. 1052), and 1% penicillin/streptomycin solution (P/S, Biochem, Cat. No. B819Q) at 37°C with 5% CO_2_ until approximately 80% confluent. Then, cells were digested with 0.25% trypsin (1:2) (Solarbio, Cat. No. P7340) for further passage.

### 2.5 Induced cell hypoxia

When HCMECs in the logarithmic growth phase reached 70%–80% confluence, the cell culture bottle was placed in a sealed plastic container, but the bottle opening was loosened to ensure gas exchange. The container was then filled with the mixed gas that induced cell hypoxia (94% N_2_ + 5% CO_2_ + 1% O_2_) and continually inflated for 10 min. The plastic container was then sealed (the bottle opening was not tightened) and placed in a 37°C incubator. HCMECs were incubated under hypoxic conditions for 1, 2, 4, 8, 12 and 24 h at different time points according to the experimental design.

### 2.6 Construction of Ang‐2 knockdown model in vitro

The gene sequence of human Ang‐2 (NM_001147.3) was first obtained by searching the NCBI website, and three target gene sequences of Ang‐2 shRNA used in this study was designed and synthesized by Zolgene Biotechnology Co., Ltd. The best interference effect of Ang‐2 knockdown were dectected by qRT‐PCR. The related sequences of Ang‐2 shRNA were presented in Table 2.

### 2.7 Cell transfection

Prior to transfection, cultured cells were seeded into 6‐well plates that were incubated in an incubator. Transfection was performed when cells reached approximately 50% confluence as assessed by inverted microscopy, and the medium without antibiotics was changed the day before transfection. With Rfect transfection reagent (Bioline, Cat. No. 11013), Ang‐2 overexpression plasmid and its negative control plasmid, Ang‐2 shRNA and its negative control plasmid, miR‐221‐3p mimics and its negative control (mimic NC), miR‐221‐3p inhibitors and its negative control (inhibitor NC) were transfected into HCMECs, respectively. miR‐221‐3p mimics, mimic NC, miR‐221‐3p inhibitors and inhibitor NC were customized from Suzhou Genepharma Co., Ltd.. Transfection efficiency and the best interference effect of Ang‐2 were determined by qRT‐PCR.

### 2.8 Cell Counting Kit (CCK)‐8 cell proliferation assay

When the density of HCMECs was about 90%, they were digested and counted. Adjust the cell density to 2 × 10^4^/mL, 100 μL of cell suspension (2 k HCMECs per well) were seeded into 96‐well plates and precultured in incubators supplemented with 5% CO_2_ at 37°C for 24 h. Before transfection, the cell culture supernatant was removed and 80 μL of complete medium was added into each well. 1.2 pmol micro‐RNA and 0.4 μL Rfect transfection reagent were diluted with 10 μL of serum‐free medium and mixed separately. After incubation at room temperature for 5 min, micro‐RNA dilution was mixed with Rfect dilution (total volume 20  μL) and incubated at room temperature for 20 min. The mixture was added into the cell culture wells to a final volume of 100 μL per well (the concentration of miR‐221‐3p mimic or miR‐221‐3p inhibitor was equivalent to 12 pmoL/mL) and incubated in a 37°C incubator. After transfection for 48 h, HCMECs were incubated at 1% O_2_ for 12, 24, 36, 48 and 72 h, respectively. Then, 10 μL of CCK‐8 solution (Solarbio, Cat. No. CA1210) and 90 μL of serum‐free medium was added to the culture plates to a final concentration of 9.6 μg/mL and incubated at 37°C for 2 h. The absorbance at 450 nm optical density (OD) value was measured using a microplate reader (Thermo Fisher Scientific). Each group was specifically designed with five parallel wells to obtain the mean value, and this current experiment was repeated a total of three times.

### 2.9 Tube formation assay

The tube formation assay was performed in fibrin matrices as described previously.^30^ Briefly, HCMECs in 100 mm dish were digested and seeded into 6‐well plates (without Matrigel) and then incubated overnight at 37°C. After cell confluence reached 50%, HCMECs were transfected with miR‐221‐3p mimic or inhibitor (final concentration 12 pmoL/mL) using Rfect transfection reagent. In the meantime, 6‐well plates were pre‐cooled overnight at 4°C. And Matrigel (BD, Cat. No. 356234) was thawed at 4°C overnight before being placed in a 6‐well plate (200 μL Matrigel/well) and incubated at 37°C for 45 min to allow solidification. After transfection for 48 h, HCMECs were treated with hypoxia (1%O_2_) for 4 h before being harvested and resuspended. 30,000 cells in 2 mL medium were seeded onto the Matrigel‐coated culture well, followed by incubation at 37°C for 8 h to promote tube formation. The tube networks were photographed from three randomly chosen fields with a microscope (Olympus Corporation) to obtain the mean value. The results were analysed using Image‐Pro Plus 6.0 software (Mshot Technology Co., Ltd). This experiment was repeated three times.

### 2.10 Grouping and identification of Ang‐2 overexpression plasmids

To assess the transfection effect of the Ang‐2 overexpression, we transfected HCMECs with Ang‐2 overexpression plasmids and negative control plasmids. Then we divided the HCMECs into the normal control group (without plasmids transfection), Ang‐2 group and Ang‐2 NC group. The Ang‐2 overexpression plasmid was constructed by Zolgene Biotechnology Co., Ltd as follows: the full‐length sequence of Ang‐2 (GENE_ID:285, RefSeq ID:NM_001147.3) was amplified by PCR in vitro, the amplified product was double digested with NheI/BamHI, and the target gene fragment was cloned into the vector pcDNA3.1(+) (Zolgene Biotechnology Co., Ltd, Cat. No. #ZVE1019) using T4 ligase to construct the recombinant Ang‐2 overexpression plasmid. HCMECs were transfected with Ang‐2 overexpression plasmids for 48 hours, and cells were collected for qRT‐PCR detection to assess Ang‐2 expression.

### 2.11 qRT‐PCR

Total RNA was isolated from HCMECs extracts using Trizol reagent according to the manufacturer's protocol (Tiangen, Cat. No. DP424). The quality of isolated RNA was assessed regarding integrity and purity of RNA. In detail, the integrity of the RNA was detected by a 1.0%–1.5% agarose gel electrophoresis. However, RNA purity were measured using an ultramicrospectrophotometer (Nanodrop 2000, Thermo Electron Corporation). According to the manufacturer's instructions. RNA purity was determined spectrophotometrically by measuring the absorbance (A) at 260 and 280 nm. The A260/A280 ratio of RNA samples was between 1.9 and 2.1, and the brightness of 28S band was twice that of 18S, which proved the quality of extracted RNA was good. Then, cDNA was synthesized with the RevertAid First Strand cDNA Synthesis Kit (Thermo, Cat. No.#K1622) from 1 μg of RNA. A total of 40 cycles were performed with the reaction system according to the manufacturer's instructions (Roche), and the cycling parameters were as follows: initial activation at 95°C for 5 min, denaturation at 94°C for 15 s, annealing at 56°C for 30 s, and extension at 72°C for 20 s (FastStart Universal SYBR Green Master (Rox), Roche, Cat. No. 04913914001). The quality of PCR products was evaluated by generating a melting curve, which was also used to verify the absence of PCR artefacts (primer dimers) or non‐specific PCR products. The small nucleolar RNA RNU6B (U6) was used as an internal reference for micro‐RNAs while the glyceraldehyde‐3‐phosphate dehydrogenase (GAPDH) was used as an internal reference for Ang‐2, CD34, insulin‐like growth factor receptor 2 (IGF1R), insulin‐like growth factor 2 (IGF‐2) and VEGFR2. The relative expression was analysed by the 2^−ΔΔCt^ method.^31^ Three parallel wells in each group were performed and an average was calculated to determine difference between the groups. All primers were purchased from Wuhan Qingke Innovation Biotechnology Co., Ltd. and used for qRT‐PCR are listed in Table 1. All experiments were repeated three times.

**TABLE 1:** Primers used for qRT‐PCR.

Primer name	Primer sequences(5′‐3′)
miR‐221‐3p‐F	GACTAGCTACATTGTCTGCTG
miR‐221‐3p‐R	GTCGTATCCAGTGCAGGGTCCGAGGTATTCGCACT GGATACGACGAAACC
miR‐222‐3p‐F	TCGGCAGGAGCTACATCTGGC
miR‐222‐3p‐R	CTCAACTGGTGTCGTGGAGTCGGCAATTCAGTTGAGACCCAGTA
U6‐F	CTCGCTTCGGCAGCACA
U6‐R	AACGCTTCACGAATTTGCGT
GAPDH‐F	GGAGCGAGATCCCTCCAAAAT
GAPDH‐R	GGCTGTTGTCATACTTCTCATGG
Ang‐2‐F	TGGCCGCAGCCTATAACA
Ang‐2‐R	TCTCTGGCAGGAGGAAAGTGT
CD34‐F	TGGGCGAAGACCCTTATTACA
CD34‐R	CGGTCCCGTTTTCCTGAG
IGF2‐F	AGCCGTGGCATCGTTGAG
IGF2‐R	ACGGGGTATCTGGGGAAGT
IGF1R‐F	CTGAAAGGAAGCGGAGAGATG
IGF1R‐R	CCGGGTCGGTGATGTTGT
VEGFR2‐F	GCCCAGGCTCAGCATACAA
VEGFR2‐R	TATTGGGCCAAAGCCAGTC

**TABLE 2:** Target gene sequence of Ang‐2 shRNA.

Sequence gene	Target sequence (5′‐3′)
Ang‐2 shRNA 1 ‐F	GATCCGGAAGAGCATGGACAGCATAGCTCGAGCTATGCTGTCCATGCTCTTCCTTTTTGC
Ang‐2 shRNA 1‐R	GGCCGCAAAAAGGAAGAGCATGGACAGCATAGCTCGAGCTATGCTGTCCATGCTCTTCCG
Ang‐2 shRNA 2‐F	GATCCGCATCTACACGTTAACATTCCCTCGAGGGAATGTTAACGTGTAGATGCTTTTTGC
Ang‐2 shRNA 2‐R	GGCCGCAAAAAGCATCTACACGTTAACATTCCCTCGAGGGAATGTTAACGTGTAGATGCG
Ang‐2 shRNA 3‐F	GATCCGCATCAGCCAACCAGGAAATGCTCGAGCATTTCCTGGTTGGCTGATGCTTTTTGC
Ang‐2‐shRNA3‐R	GGCCGCAAAAAGCATCAGCCAACCAGGAAATGCTCGAGCATTTCCTGGTTGGCTGATGCG

### 2.12 Western blotting

Western blotting was performed as described previously by Zhang, et al.^32^ Briefly, protein was extracted from HCMECs using 100 μL of precooled protein lysis buffer per well of 6‐well plate (0.1 M NaCl, 10 mM Tris–HCl (pH 8.0), 1 mM EDTA, 100 μg/mL phenylmethylsulphonylfluoride (PMSF), 1% NP40, 1/100 protease inhibitor cocktail, and 1/100 phosphatase inhibitor cocktail (Sigma‐Aldrich)). Cell lysates were placed on ice with agitation for 30 min and then sonicated for 10 s at 30 W using Ultrasonic Homogenizer (Ningbo Scientz Biotechnology Co., Ltd.). Cell lysates were spinned at 10,000 g for 5 min at 4°C to remove insoluble substances. Protein quantification was performed using Bicinchoninic Acid (BCA) method, and the absorbance at 562 nm was measured by UV spectrophotometer. Equal amounts of protein (40 μg) were seperated in 10% gel electrophoresis (SDS‐PAGE). The proteins were then transferred to a polyvinylidene difluoride (PVDF) membrane for 2 h at 200 V using a wet transfer system. Then, the membrane was blocked for 1 hour at room temperature with 5% BSA in tris‐buffered saline containing 0.1% tween (TBS‐T), followed by overnight incubation at 4°C with primary antibodies. Horseradish peroxidase‐conjugated secondary antibodies were used to locate the primary antibodies with the enhanced chemoluminescence (ECL) kit (Yeasen, Cat. No. 36208ES76). Bands were quantified by densitometric analysis using the Image software (Bio‐Rad) and normalized against GAPDH signal for total protein assessment. All primary antibodies including anti‐Ang‐2 antibody (Abcam, Cat. No. EPR2891 (2)), Anti‐IGF2 antibody (Abcam, Cat. No. EPR12221), Anti‐IGF1 Receptor antibody (Abcam, Cat. No. EPR23027‐204), Anti‐VEGF Receptor2 antibody (Abcam, Cat. No. EPRER16Y), and Anti‐GAPDH antibody (Wuhan Cloud Clone Technology Co., Cat. No. PAB932Hu01) were diluted by 1:1000 except for that for Anti‐CD34 antibody (Abcam, Cat. No. EPR2999) by 1:50000. All experiment were repeated a total of three times.

### 2.13 Statistical analysis

All statistical analyses were performed using SPSS version 20.0 software (IBM Corporation) and GraphPad Prism version 8.0.1 software. Data are presented as the mean ± SEM. Student's *t*‐test was used for data with two groups while one‐way anova was used for data with more than two groups. The SNK‐Q or LSD test was used for comparisons between groups when variances were homogeneous, and the Games Howell test was used for comparisons when variances were not homogeneous. *p value* <0. 05 was considered statistically significant (*p < 0.05, **p < 0.01).

## 3 RESULTS

### 3.1 Effects of hypoxia on miR‐221‐3p expression in HCMECs

**FIGURE 1:**
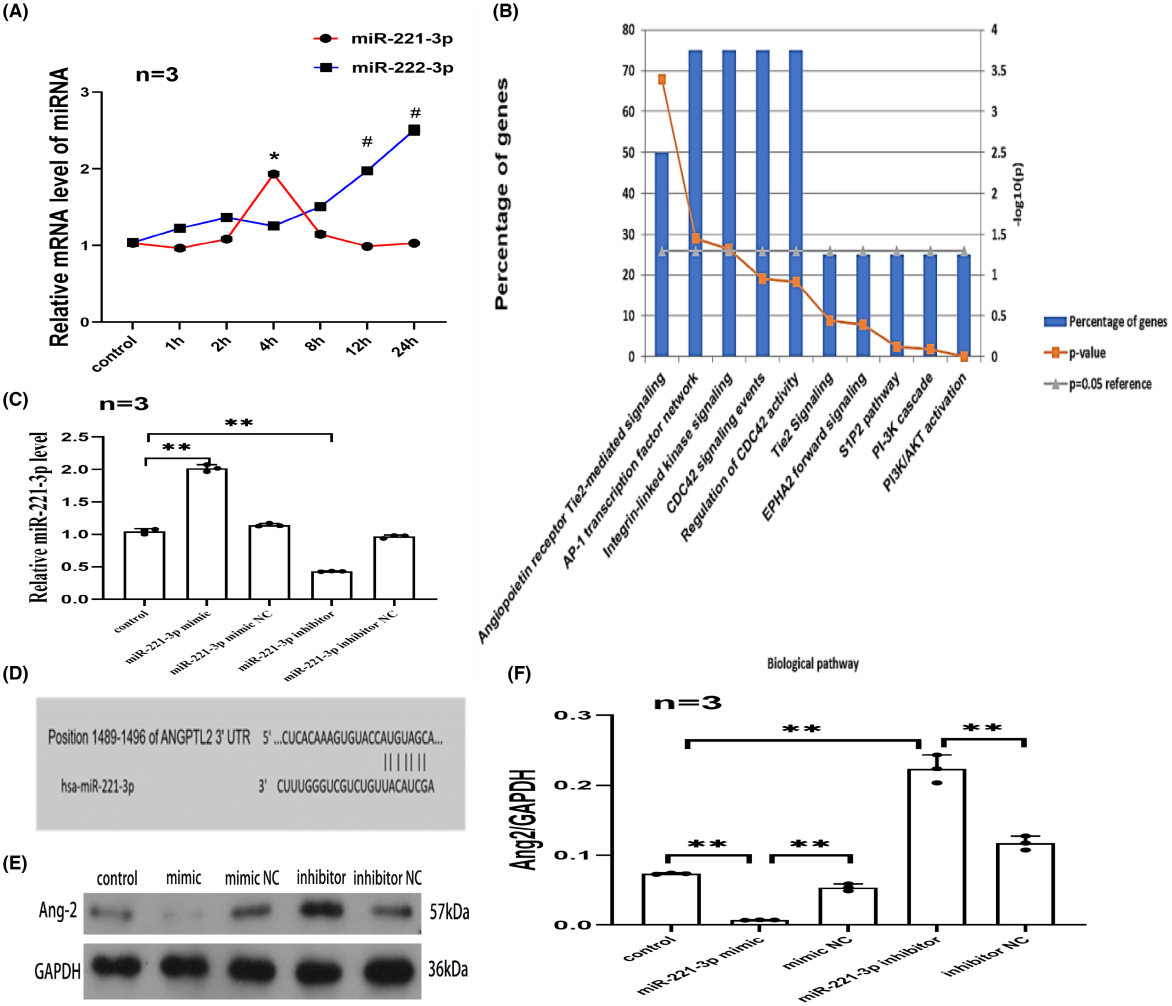
miR‐221‐3p targets Ang‐2 and inhibits Ang‐2 expression under hypoxic conditions.

qPCR was performed to detect miR‐221‐3p and miR‐222‐3p expression in HCMECs at different time points (1, 2, 4, 8, 12 and 24 h) under hypoxic conditions (1% O_2_). As shown in Figure 1A, miR‐221‐3p was significantly increased when HCMECs were exposed to hypoxia for 4 h. Although miR‐222‐3p expression level was significantly elevated at 12 and 24 h of hypoxia in HCMECs, miR‐222‐3p was not significantly altered when HCMECs were exposed to hypoxia for 4 h. Therefore, to further explore the role of miR‐221‐3p in angiogenesis and avoid interference of miR‐222‐3p, HCMECs were cultured in an atmosphere of 1% O_2_ for 4 h prior to subsequent cells transfection.

### 3.2 miR‐221‐3p inhibited Ang‐2 expression in HCMECs under hypoxic conditions

Funrich3.13 software was used to enrich the target genes of miR‐221‐3p that have been validated in previous publications. It was discovered that miR‐221‐3p was predominantly associated with the angiopoietin receptor‐Tie2 pathway (Figure 1B). Then, we used TargetScan 7.2 to predict the target gene, and our results revealed that there were binding sites between miR‐221‐3p and Ang‐2 (Figure 1D). To further demonstrate the regulatory link between miR‐221‐3p and Ang‐2, HCMECs were transfected with miR‐221‐3p mimic and inhibitor, resulting in miR‐221‐3p overexpression and knockdown, respectively (Figure 1C). The western blot method was used to detect the level of Ang‐2 expression. Overexpression of miR‐221‐3p reduced Ang‐2 expression; whereas, miR‐221‐3p knockdown led to increased Ang‐2 expression (Figure 1E,F). These data imply that miR‐221‐3p suppresses Ang‐2 expression under hypoxia by targeting Ang‐2.

### 3.3 miR‐221‐3p inhibited HCMECs angiogenesis in vitro under hypoxic conditions

**FIGURE 2:**
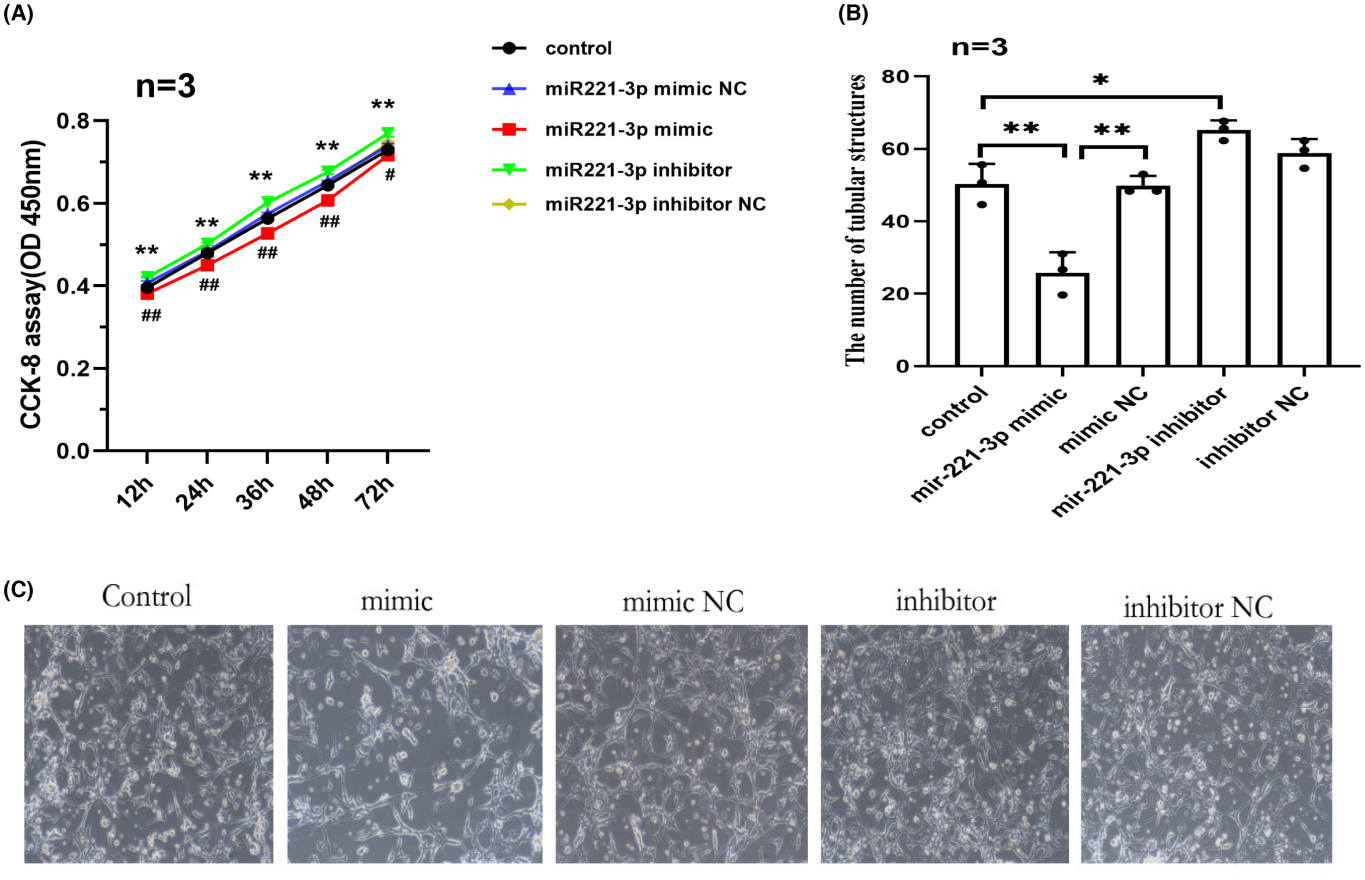
miR‐221‐3p inhibited HCMECs angiogenesis in vitro under hypoxic conditions.

To determine the effect of miR‐221‐3p on the angiogenesis of HCMECs, we transfected hypoxia‐treated HCMECs with miR‐221‐3p mimics and inhibitors in vitro. CCK‐8 assay results showed that miR‐221‐3p overexpression inhibited HCMECs proliferation; whereas, miR‐221‐3p inhibition promoted proliferation (Figure 2A). Then, tube formation was assessed in hypoxia‐treated HCMECs transfected with miR‐221‐3p mimics and inhibitors. Compared with the control group and miR‐221‐3p mimic NC group, the number of tubular structures was significantly decreased in the miR‐221‐3p mimic group. Therefore, miR‐221‐3p overexpression inhibited tube formation, whereas inhibition of miR‐221‐3p expression had the reverse effect (Figure 2B,C).

### 3.4 miR‐221‐3p inhibited CD34, IGF1R, IGF‐2 and VEGFR2 proteins expression in HCMECs under hypoxic conditions

**FIGURE 3:**
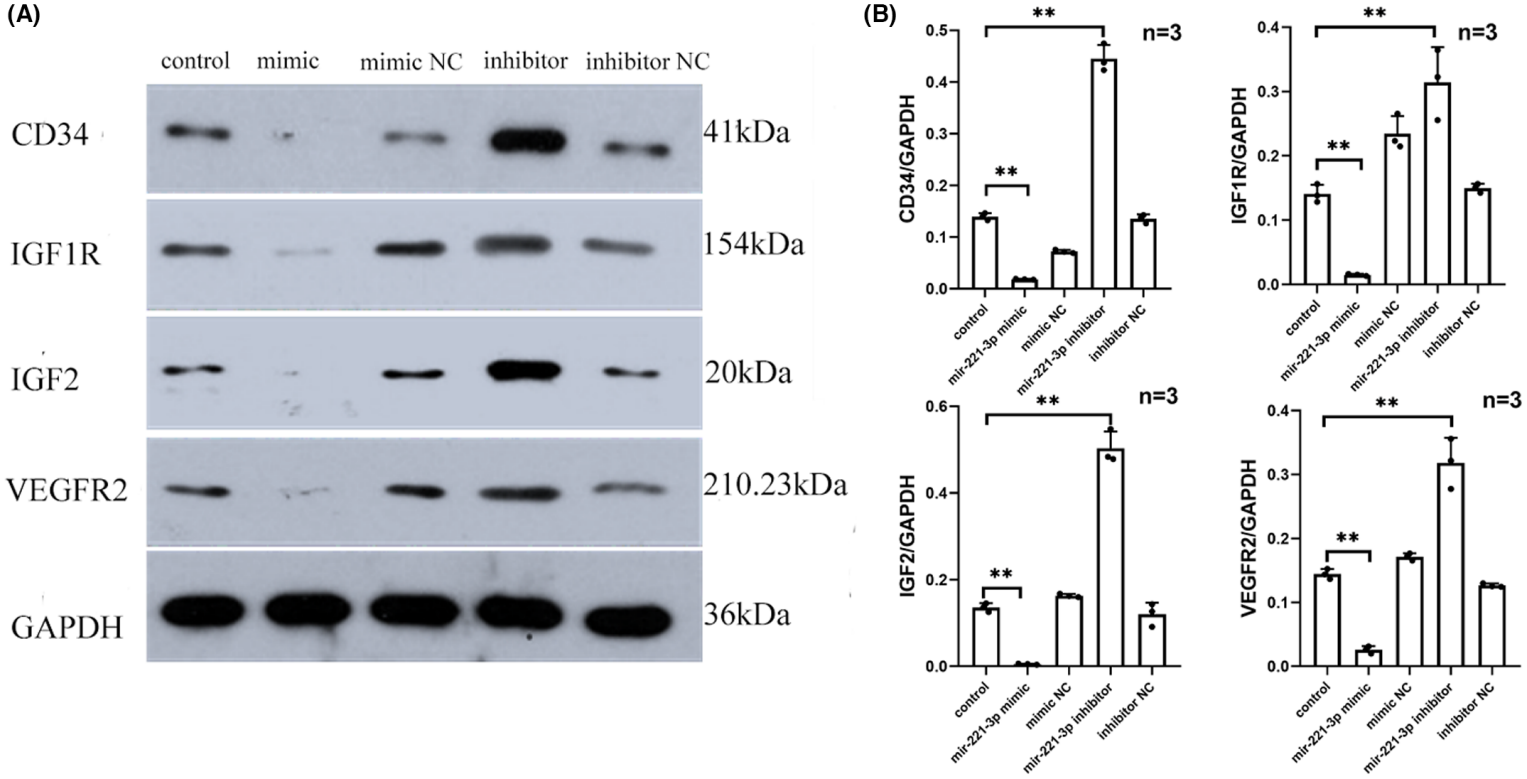
miR‐221‐3p inhibited the expression of tip cell marker genes in vitro under hypoxic conditions.

Following hypoxia induction in HCMECs in vitro, cells were transfected with miR‐221‐3p mimic, miR‐221‐3p inhibitor and negative controls (miR‐221‐3p mimic NC and miR‐221‐3p inhibitor NC) according to the assay design. CD34, IGF1R, IGF‐2 and VEGFR2 protein expression levels were considerably lower in the miR‐221‐3p mimic group than in the control group and the miR‐221‐3p mimic NC group, as determined by western blotting (*p* < 0.05). In contrast, compared to the control and miR‐221‐3p inhibitor NC groups, the expression levels of CD34, IGF1R, IGF‐2 and VEGFR2 proteins were considerably increased in the miR‐221‐3p inhibitor group (*p* < 0.05).Therefore, the transfection of miR‐221‐3p under hypoxia inhibited the expression of CD34, IGF1R, IGF‐2 and VEGFR2 proteins in HCMECs under hypoxic conditions (Figure 3A,B).

### 3.5 Identification of the transfection effect of the Ang‐2 overexpression plasmid

**FIGURE 4:**
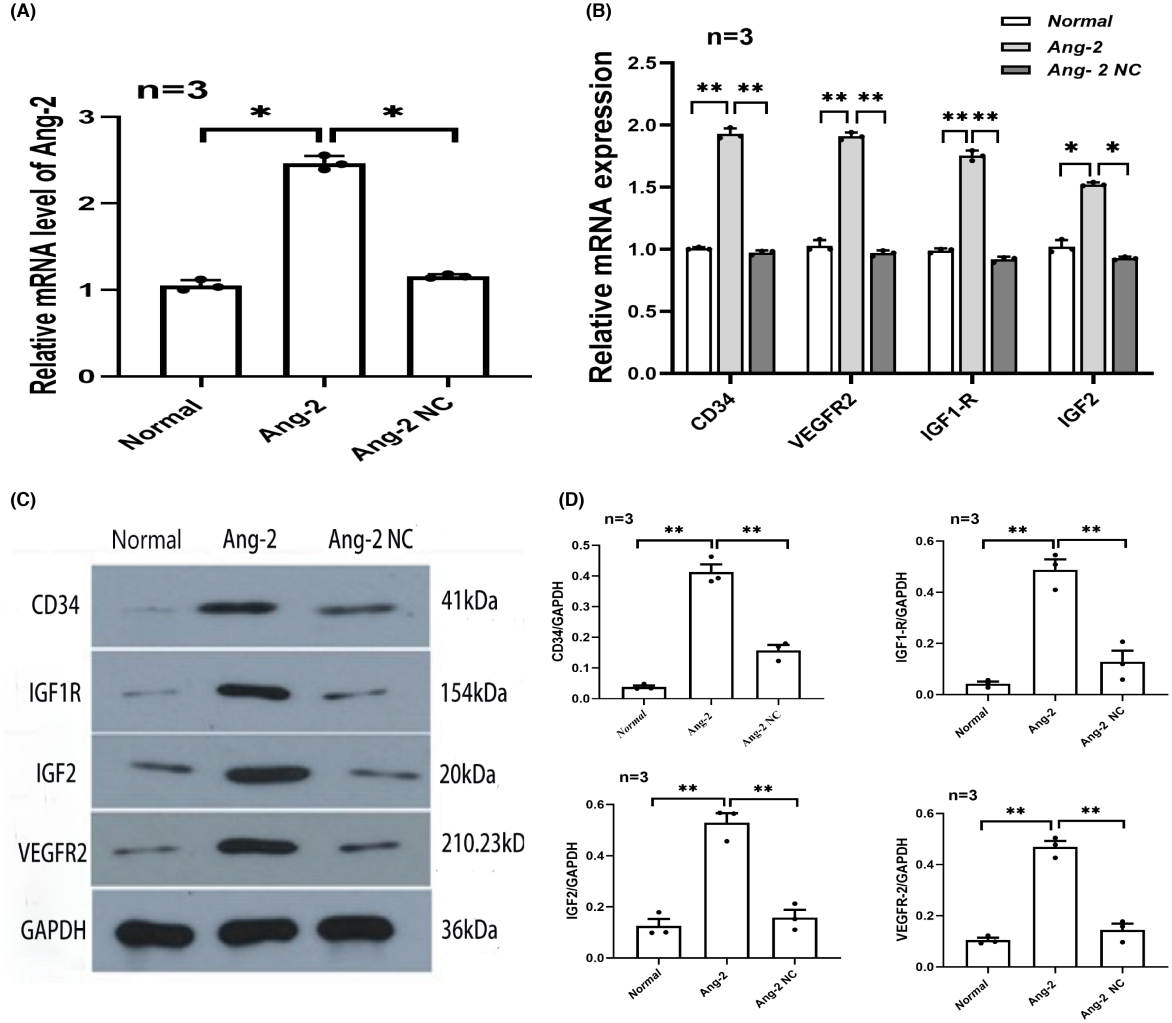
Ang‐2 promoted CD34, IGF1R, IGF‐2 and VEGFR2 expression in vitro under hypoxic conditions.

The qRT‐PCR results revealed that Ang‐2 mRNA expression in the Ang‐2 group was significantly higher than that in the normal group and Ang‐2 NC group (*p* < 0.05). This result demonstrated that the Ang‐2 overexpression plasmid transfection was successful (Figure 4A).

### 3.6 Ang‐2 induced CD34, IGF1R, IGF‐2 and VEGFR2 expression in HCMECs under hypoxic conditions

To clarify the relationship between Ang‐2 and CD34, IGF1R, IGF‐2 and VEGFR2 expression in HCMECs after induction of hypoxia, HCMECs were transfected with the Ang‐2 overexpression plasmid under hypoxic conditions. After transfection with the Ang‐2 overexpression plasmid, qRT‐PCR results showed that CD34, IGF1R, IGF‐2 and VEGFR2 mRNA expression levels were significantly increased in the Ang‐2 group compared with the normal group and Ang‐2 NC group (*p* < 0.01) (Figure 4B). Moreover, western blotting results showed that CD34, IGF1R, IGF‐2 and VEGFR2 proteins expression levels were significantly increased in the Ang‐2 group compared with the normal group and Ang‐2 NC group (*p* < 0.01) (Figure 4C,D).

### 3.7 Ang‐2 knockdown overturned the effect of miR‐221‐3p inhibitor in promoting CD34, IGF1R, IGF‐2 and VEGFR2 expression in HCMECs under hypoxic conditions

**FIGURE 5:**
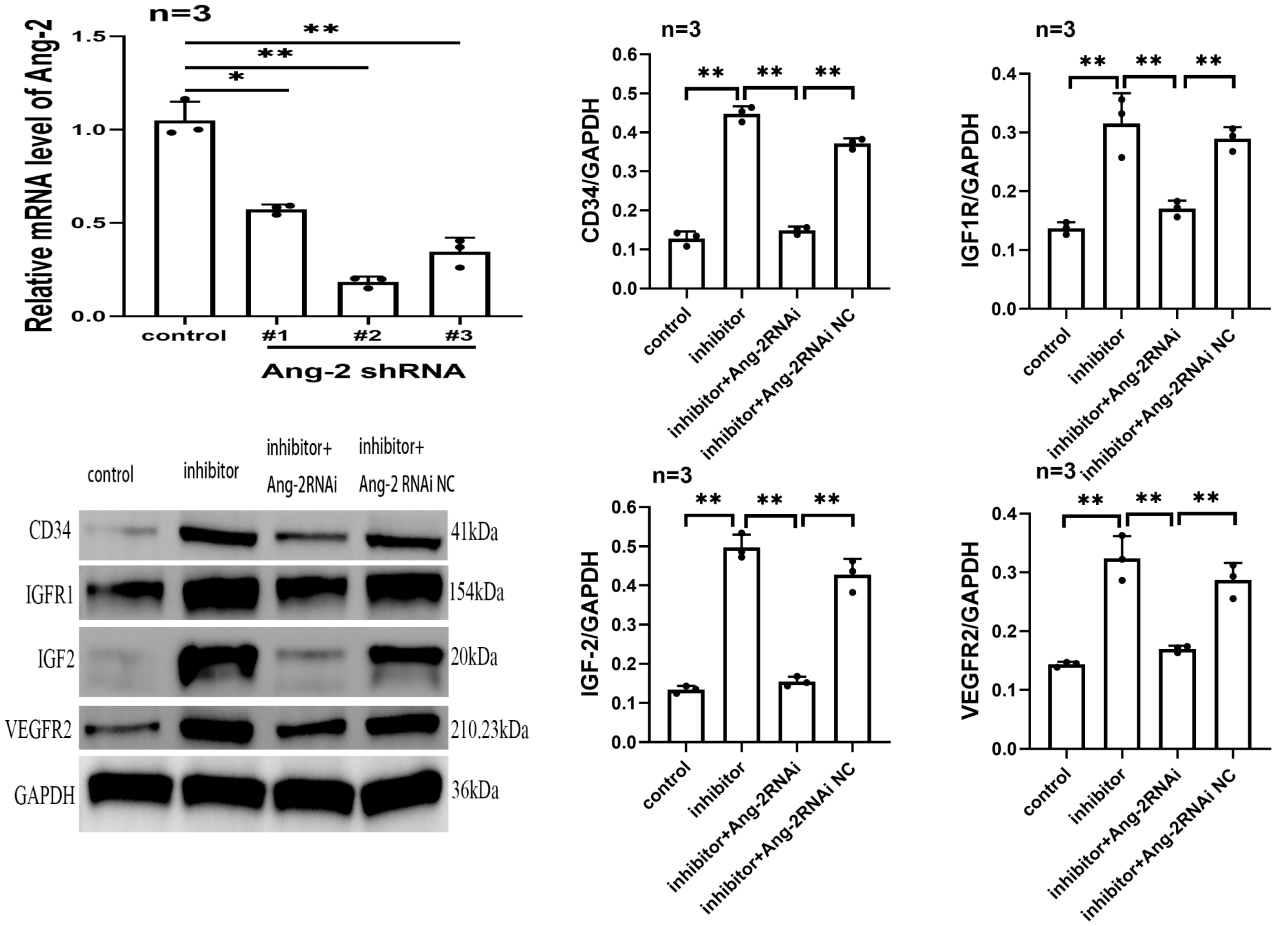
Ang‐2 knockdown overturned the effect of miR‐221‐3p inhibitor in promoting CD34, IGF1R, IGF‐2 and VEGFR2 proteins expression in HCMECs under hypoxic conditions.

HCMECs were cultured under normoxic conditions (95% air, 5% CO_2_, 37°C). qRT‐PCR results showed that the Ang‐2 mRNA expression in HCMECs of shRNA‐2 group was significantly lower than that of shRNA‐1 group, shRNA‐3 group and normal control group, indicating that Ang‐2 shRNA‐2 had the best interference effect (Figure 5A). Therefore, it was chosed as the interfering fragment for further experiments. In order to clarify the dominant role of Ang‐2 in the promoting effect of miR‐221‐3p inhibitor, miR‐221‐3p inhibitor‐treated HCMECs were transfected by Ang‐2 shRNA under hypoxic conditions. The results revealed that the protein expression levels of CD34, IGFR1, IGF‐2 and VEGFR2 in HCMECs under hypoxic conditions were significantly decreased after Ang‐2 shRNA‐2 transfection (*p* < 0.01) (Figure 5B,C). Therefore, Ang‐2 knockdown significantly overturned the effect of miR‐221‐3p inhibitor in promoting the transformation of HCMECs to tip cells.

### 3.8 miR‐221‐3p expression level was increased in the acute myocardial infarction model

**FIGURE 6:**
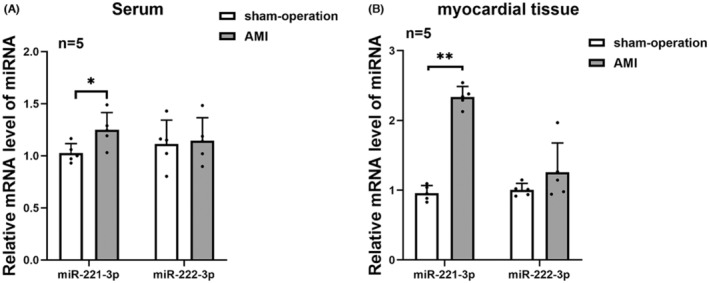
The mNRA expression level of miR‐221‐3p and miR‐222‐3p in acute myocardial infarction model.

In order to initially understand the role of miR‐221‐3p in angiogenesis after acute myocardial infarction, we constructed a preclinical model of acute myocardial infarction by ligating the LAD coronary artery. The results revealed that the expression level of miR‐222‐3p mRNA in both serum and myocardial tissues of rats in AMI group was not significantly elevated compared with the sham‐operation group. However, the expression level of miR‐221‐3p mRNA was significantly increased in both serum and myocardial tissues of rats with acute myocardial infarction (*p* < 0.01) (Figure 6A,B).

## 4 DISCUSSION

Angiogenesis is a highly coordinated tissue remodelling process that leads to vascularization. Angiogenesis is often triggered by an imbalance between oxygen consumption and demand, and the mechanisms of hypoxia‐induced angiogenesis have been intensively studied over the past decade.^33^, ^34^, ^35^ With the booming biomedical research in the field of miRNAs, the role of miRNAs in angiogenesis is being thoroughly studied. And miR‐221 and miR‐222 are two highly homologous miRNAs that both have a role in regulating angiogenesis. However, by analysing the transcriptome profile of HCMECs under hypoxic conditions, we only observed high expression of miR‐221‐3p in HCMECs 4 h after induction of hypoxia (1% O_2_) while there was no statistically significant difference in miR‐222‐3p expression level after induction of hypoxia for 4 h. Moreover, our cell proliferation assay results indicated miR‐221‐3p inhibited endothelial cell proliferation and tube formation. Thus, we propose that miR‐221‐3p plays an important role in hypoxia‐induced angiogenesis due to its involvement in the major steps of angiogenesis.

Ang‐1 and Ang‐2 are both members of the growth factor family and play important roles in angiogenesis. Many reports have shown that Ang‐2 is a high affinity ligand for the tie2 receptor that negatively affects vascular stabilization and maturation by antagonizing Ang‐1/tie2.^36^, ^37^, ^38^ However, it has also been suggested that Ang‐2 can activate endothelial cells and exert proangiogenic effects under certain conditions.^39^, ^40^, ^41^ Rational use of agents targeting Ang‐2 yield limited efficacy as antiangiogenic interventions due to this paradoxical effect.^38^ In our study, the results of TargetScan 7.2 supported the targeting of miR‐221‐3p to Ang‐2, demonstrating that Ang‐2 is one of the downstream targets of miR‐221‐3p. Moreover, miR‐221‐3p overexpression reduced Ang‐2 protein expression in HCMECs under hypoxic conditions, inhibiting the proliferation and tube formation of HCMECs. In contrast, transfection of miR‐221‐3p inhibitor yielded the opposite results. Therefore, miR‐221‐3p can target Ang‐2 and downregulate Ang‐2 levels to inhibit angiogenesis.

Tip cells are a subset of endothelial cells with distinct molecular features and exhibit an endothelial transdifferentiation phenotype.^3^ As an essential pathway for angiogenesis, tip cell formation is an attractive target for proangiogenic and antiangiogenic therapies.^42^ Siemerink, et al. identified tip cells in HUVECs culture using CD34 as a marker.^42^ Moreover, they identified CD34 and VEGFR2 as the most significantly differentially expressed marker genes in the tip cells. Both IGF‐2 and IGF1R are novel marker genes for tip cells, and IGF‐2 and IGF1R proteins are required for the maintenance of the tip cell phenotype.^3^ Based on the above findings, the present experiment also detected the corresponding protein expression of marker genes, including CD34, VEGFR2, IGF1R and IGF‐2, which serve as markers of the transformation of HCMECs to tip cells and the formation of tip cells. Our study found that miR‐221‐3p overexpression reduced CD34, VEGFR2, IGF1R and IGF‐2 protein levels in HCMECs under hypoxic conditions; whereas, transfection of miR‐221‐3p inhibitor increased protein expression levels of CD34, VEGFR2, IGF1R and IGF‐2 under hypoxic conditions. Moreover, Ang‐2 overexpression can lead to increased CD34, VEGFR2, IGF1R and IGF‐2 protein and mRNA expression levels under hypoxic conditions. To further clarify that Ang‐2 plays a dominant role in miR‐221‐3p inhibitors promoting the transformation of HCMECs to tip cells, we used Ang‐2 shRNA to interfere with miR‐221‐3p inhibitor‐treated HCMECs under hypoxic conditions. As we expected, Ang‐2 knockdown significantly reduced the promoting effect of miR‐221‐3p inhibitor. Hence, miR‐221‐3p exhibits antiangiogenic properties by targeting Ang‐2 to inhibit the transformation of HCMECs into tip cells under hypoxic conditions.

Acute myocardial infarction (AMI) is an ischemic myocardial necrosis event caused by acute coronary artery occlusion, which is one of the leading causes of death in adults worldwide.^43^ Currently, ischemia‐reperfusion therapy is the best treatment strategy to save ischemic myocardium and improve cardiac function in AMI patients. In addition to ischemia‐reperfusion therapy, promoting angiogenesis is also an urgent and necessary for ameliorating the adverse prognosis of AMI patients.^44^ We constructed an AMI rat model by ligating the LAD coronary artery and found that the mRNA expression level of miR‐221‐3p was significantly elevated both in the serum and myocardial tissue of AMI rats. Nevertheless, there was no significant difference in the mRNA expression level of miR‐222‐3p in serum and myocardial tissue of rats in the AMI group compared with the sham‐operated group. Combined with our cellular experiments' results, miR‐221‐3p overexpression may inhibits angiogenesis after acute myocardial infarction by targeting Ang‐2 to inhibit the transformation of endothelial cells to tip cells, which needs to be further confirmed in animal models.

## 5 CONCLUSION

In conclusion, this is the first report demonstrating that miR‐221‐3p negatively regulates angiogenesis by targeting Ang‐2, expanding our knowledge of miRNAs and providing novel insights on therapeutic strategies for antiangiogenesis. Inhibiting miR‐221‐3p may promote angiogenesis, cardiac repair and myocardial survival after acute myocardial infarction by targeting Ang‐2.

## AUTHOR CONTRIBUTIONS

**Peng Yang:** Conceptualization (equal); data curation (equal); writing – original draft (lead). **Qing Yang:** Conceptualization (equal); data curation (equal). **Yiheng Yang:** Formal analysis (equal). **Qingshan Tian:** Formal analysis (equal). **Zhenzhong Zheng:** Conceptualization (equal); data curation (equal); funding acquisition (lead); writing – review and editing (lead).

## FUNDING INFORMATION

This work was supported by grants from the 2022 Senior Science and Technology Innovation Talent Program of Nanchang City (2022‐321‐13), the 2020 Natural Science Foundation of Jiangxi Province in China (20202ABCL206002), and the key laboratory of nanchang city (2021‐NCZDSY‐009).

## CONFLICT OF INTEREST STATEMENT

The authors declare that they have no conflicts of interest.

## Data Availability

The data that support the findings of this study are available from the corresponding author upon reasonable request.

## References

[1] Wang H , Cai J . The role of microRNAs in heart failure. Biochimica et Biophysica Acta Molecular Basis of Disease. 2017;1863(8):2019‐2030. 27916680 10.1016/j.bbadis.2016.11.034

[2] Potente M , Gerhardt H , Carmeliet P . Basic and therapeutic aspects of angiogenesis. Cell. 2011;146(6):873‐887. 21925313 10.1016/j.cell.2011.08.039

[3] Dallinga MG , Yetkin‐Arik B , Kayser RP , et al. IGF2 and IGF1R identified as novel tip cell genes in primary microvascular endothelial cell monolayers. Angiogenesis. 2018;21(4):823‐836. 29951828 10.1007/s10456-018-9627-4 PMC6208896

[4] Bikfalvi A . History and conceptual developments in vascular biology and angiogenesis research: a personal view. Angiogenesis. 2017;20(4):463‐478. 28741165 10.1007/s10456-017-9569-2

[5] Fong GH . Mechanisms of adaptive angiogenesis to tissue hypoxia. Angiogenesis. 2008;11(2):121‐140. 18327686 10.1007/s10456-008-9107-3

[6] Arora S , Rana R , Chhabra A , Jaiswal A , Rani V . miRNA‐transcription factor interactions: a combinatorial regulation of gene expression. Molecular Genetics and Genomics. 2013;288(3–4):77‐87. 23334784 10.1007/s00438-013-0734-z

[7] Schober A , Weber C . Mechanisms of microRNAs in atherosclerosis. Annu Rev Pathol. 2016;11:583‐616. 27193456 10.1146/annurev-pathol-012615-044135

[8] Lu D , Thum T . RNA‐based diagnostic and therapeutic strategies for cardiovascular disease. Nat Rev Cardiol. 2019;16(11):661‐674. 31186539 10.1038/s41569-019-0218-x

[9] Trabucchi M , Mategot R . Subcellular heterogeneity of the microRNA machinery. Trends Genet. 2020;36(1):70. 31597611 10.1016/j.tig.2019.07.008

[10] Wu YY , Chen YL , Jao YC , Hsieh IS , Chang KC , Hong TM . miR‐320 regulates tumor angiogenesis driven by vascular endothelial cells in oral cancer by silencing neuropilin 1. Angiogenesis. 2014;17(1):247‐260. 24114198 10.1007/s10456-013-9394-1

[11] Ohyagi‐Hara C , Sawada K , Kamiura S , et al. miR‐92a inhibits peritoneal dissemination of ovarian cancer cells by inhibiting integrin α5 expression. Am J Pathol. 2013;182(5):1876‐1889. 23499550 10.1016/j.ajpath.2013.01.039

[12] Shi ZM , Wang J , Yan Z , et al. MiR‐128 inhibits tumor growth and angiogenesis by targeting p70S6K1. PLoS One. 2012;7(3):e32709. 22442669 10.1371/journal.pone.0032709 PMC3307714

[13] Jakob P , Doerries C , Briand S , et al. Loss of angiomiR‐126 and 130a in angiogenic early outgrowth cells from patients with chronic heart failure: role for impaired in vivo neovascularization and cardiac repair capacity. Circulation. 2012;126(25):2962‐2975. 23136161 10.1161/CIRCULATIONAHA.112.093906

[14] Fasanaro P , D'Alessandra Y , Di Stefano V , et al. MicroRNA‐210 modulates endothelial cell response to hypoxia and inhibits the receptor tyrosine kinase ligand ephrin‐A3. J Biol Chem. 2008;283(23):15878‐15883. 18417479 10.1074/jbc.M800731200 PMC3259646

[15] Duan Q , Yang L , Gong W , et al. MicroRNA‐214 is upregulated in heart failure patients and suppresses XBP1‐mediated endothelial cells angiogenesis. J Cell Physiol. 2015;230(8):1964‐1973. 25656649 10.1002/jcp.24942 PMC4911176

[16] Mayoral RJ , Pipkin ME , Pachkov M , van Nimwegen E , Rao A , Monticelli S . MicroRNA‐221‐222 regulate the cell cycle in mast cells. J Immunol. 2009;182(1):433‐445. 19109175 10.4049/jimmunol.182.1.433 PMC2610349

[17] Nicoli S , Knyphausen CP , Zhu LJ , Lakshmanan A , Lawson ND . miR‐221 is required for endothelial tip cell behaviors during vascular development. Dev Cell. 2012;22(2):418‐429. 22340502 10.1016/j.devcel.2012.01.008 PMC3285411

[18] Kir D , Schnettler E , Modi S , Ramakrishnan S . Regulation of angiogenesis by microRNAs in cardiovascular diseases. Angiogenesis. 2018;21(4):699‐710. 29956018 10.1007/s10456-018-9632-7

[19] Li Y , Yan C , Fan J , Hou Z , Han Y . MiR‐221‐3p targets Hif‐1α to inhibit angiogenesis in heart failure. Lab Invest. 2021;101(1):104‐115. 32873879 10.1038/s41374-020-0450-3

[20] Yang Y , Li H , Ma Y , Zhu X , Zhang S , Li J . MiR‐221‐3p is down‐regulated in preeclampsia and affects trophoblast growth, invasion and migration partly via targeting thrombospondin 2. Biomed Pharmacother. 2019;109:127‐134. 30396069 10.1016/j.biopha.2018.10.009

[21] He L , Dang L , Zhou J , Bai J , Li YZ . Association of angiopoietin‐1, angiopoietin‐2 and caspase‐5 polymorphisms with psoriasis vulgaris. Clin Exp Dermatol. 2015;40(5):556‐563. 25753570 10.1111/ced.12550

[22] Leong A , Kim M . The Angiopoietin‐2 and TIE pathway as a therapeutic target for enhancing antiangiogenic therapy and immunotherapy in patients with advanced cancer. Int J Mol Sci. 2020;21(22):8689. 33217955 10.3390/ijms21228689 PMC7698611

[23] Okada H , Tsuzuki T , Shindoh H , Nishigaki A , Yasuda K , Kanzaki H . Regulation of decidualization and angiogenesis in the human endometrium: mini review. J Obstet Gynaecol Res. 2014;40(5):1180‐1187. 24754847 10.1111/jog.12392

[24] Hussain R , Neiweem A , Kansara V , Harris A , Ciulla T . Tie‐2/angiopoietin pathway modulation as a therapeutic strategy for retinal disease. Expert Opin Investig Drugs. 2019;28(10):861‐869. 10.1080/13543784.2019.1667333 31513439

[25] Wu Q , Xu WD , Huang AF . Role of angiopoietin‐2 in inflammatory autoimmune diseases: a comprehensive review. Int Immunopharmacol. 2020;80:106223. 31991374 10.1016/j.intimp.2020.106223

[26] Hammes H , Lin J , Wagner P , et al. Angiopoietin‐2 causes pericyte dropout in the normal retina: evidence for involvement in diabetic retinopathy. Diabetes. 2004;53(4):1104‐1110. 15047628 10.2337/diabetes.53.4.1104

[27] Biel N , Siemann D . Targeting the Angiopoietin‐2/Tie‐2 axis in conjunction with VEGF signal interference. Cancer Lett. 2016;380(2):525‐533. 25312939 10.1016/j.canlet.2014.09.035 PMC4394020

[28] Zeng A , Wang SR , He YX , Yan Y , Zhang Y . Progress in understanding of the stalk and tip cells formation involvement in angiogenesis mechanisms. Tissue Cell. 2021;73:101626. 34479073 10.1016/j.tice.2021.101626

[29] Wang D , Tian L , Lv H , et al. Chlorogenic acid prevents acute myocardial infarction in rats by reducing inflammatory damage and oxidative stress. Biomed Pharmacother. 2020;132:110773. 33022535 10.1016/j.biopha.2020.110773

[30] Liu Y , Zou J , Li B , et al. RUNX3 modulates hypoxia‐induced endothelial‐to‐mesenchymal transition of human cardiac microvascular endothelial cells. Int J Mol Med. 2017;40(1):65‐74. 28534977 10.3892/ijmm.2017.2998 PMC5466396

[31] Livak KJ , Schmittgen TD . Analysis of relative gene expression data using real‐time quantitative PCR and the 2(‐Delta Delta C(T)) method. Methods. 2001;25(4):402‐408. 11846609 10.1006/meth.2001.1262

[32] Zhang G , Liu Z , Ding H , et al. Tumor induces muscle wasting in mice through releasing extracellular Hsp70 and Hsp90. Nat Commun. 2017;8(1):589. 28928431 10.1038/s41467-017-00726-x PMC5605540

[33] Jain RK . Antiangiogenesis strategies revisited: from starving tumors to alleviating hypoxia. Cancer Cell. 2014;26(5):605‐622. 25517747 10.1016/j.ccell.2014.10.006 PMC4269830

[34] Germain S , Monnot C , Muller L , Eichmann A . Hypoxia‐driven angiogenesis: role of tip cells and extracellular matrix scaffolding. Curr Opin Hematol. 2010;17(3):245‐251. 20308893 10.1097/MOH.0b013e32833865b9

[35] Wong BW , Marsch E , Treps L , Baes M , Carmeliet P . Endothelial cell metabolism in health and disease: impact of hypoxia. EMBO J. 2017;36(15):2187‐2203. 28637793 10.15252/embj.201696150 PMC5538796

[36] Reiss Y , Droste J , Heil M , et al. Angiopoietin‐2 impairs revascularization after limb ischemia. Circ Res. 2007;101(1):88‐96. 17540977 10.1161/CIRCRESAHA.106.143594

[37] Fagiani E , Christofori G . Angiopoietins in angiogenesis. Cancer Lett. 2013;328(1):18‐26. 22922303 10.1016/j.canlet.2012.08.018

[38] Felcht M , Luck R , Schering A , et al. Angiopoietin‐2 differentially regulates angiogenesis through TIE2 and integrin signaling. J Clin Invest. 2012;122(6):1991‐2005. 22585576 10.1172/JCI58832 PMC3366398

[39] Yuan HT , Khankin EV , Karumanchi SA , Parikh SM . Angiopoietin 2 is a partial agonist/antagonist of Tie2 signaling in the endothelium. Mol Cell Biol. 2009;29(8):2011‐2022. 19223473 10.1128/MCB.01472-08 PMC2663314

[40] Tressel SL , Huang RP , Tomsen N , Jo H . Laminar shear inhibits tubule formation and migration of endothelial cells by an angiopoietin‐2 dependent mechanism. Arterioscler Thromb Vasc Biol. 2007;27(10):2150‐2156. 17673702 10.1161/ATVBAHA.107.150920 PMC2699463

[41] An YA , Sun K , Joffin N , et al. Angiopoietin‐2 in white adipose tissue improves metabolic homeostasis through enhanced angiogenesis. Elife. 2017;6:e24071. 28355132 10.7554/eLife.24071 PMC5391203

[42] Siemerink MJ , Klaassen I , Vogels IM , Griffioen AW , Van Noorden CJ , Schlingemann RO . CD34 marks angiogenic tip cells in human vascular endothelial cell cultures. Angiogenesis. 2012;15(1):151‐163. 22249946 10.1007/s10456-011-9251-z PMC3274677

[43] Anderson J , Morrow D . Acute myocardial infarction. N Engl J Med. 2017;376(21):2053‐2064. 28538121 10.1056/NEJMra1606915

[44] Wu X , Reboll M , Korf‐Klingebiel M , Wollert K . Angiogenesis after acute myocardial infarction. Cardiovasc Res. 2021;117(5):1257‐1273. 33063086 10.1093/cvr/cvaa287

